# Improving Precise Genome Editing Using Donor DNA/gRNA Hybrid Duplex Generated by Complementary Bases

**DOI:** 10.3390/biom12111621

**Published:** 2022-11-03

**Authors:** Wataru Aiba, Takamitsu Amai, Mitsuyoshi Ueda, Kouichi Kuroda

**Affiliations:** Division of Applied Life Sciences, Graduate School of Agriculture, Kyoto University, Sakyo-ku, Kyoto 606-8502, Japan

**Keywords:** CRISPR/Cas9, genome editing, single-stranded oligodeoxynucleotide, guide RNA, DNA/RNA hybrid, *Saccharomyces cerevisiae*

## Abstract

In precise genome editing, site-specific DNA double-strand breaks (DSBs) induced by the CRISPR/Cas9 system are repaired via homology-directed repair (HDR) using exogenous donor DNA templates. However, the low efficiency of HDR-mediated genome editing is a barrier to widespread use. In this study, we created a donor DNA/guide RNA (gRNA) hybrid duplex (DGybrid) that was composed of sequence-extended gRNA and single-stranded oligodeoxynucleotide (ssODN) combined with complementary bases without chemical modifications to increase the concentration of donor DNA at the cleavage site. The efficiency of genome editing using DGybrid was evaluated in *Saccharomyces cerevisiae*. The results show a 1.8-fold (from 35% to 62%) improvement in HDR-mediated editing efficiency compared to genome editing in which gRNA and donor DNA were introduced separately. In addition, analysis of the nucleic acid introduction efficiency using flow cytometry indicated that both RNA and ssODNs are efficiently incorporated into cells together by using the DNA/RNA hybrid. Our technique would be preferred as a universal and concise tool for improving the efficiency of HDR-mediated genome editing.

## 1. Introduction

The CRISPR/Cas9 system, a powerful method for genome editing, is rapidly being improved by researchers [1,2]. The system is used for a wide range of biomedical and biotechnological purposes, including the repair of undesirable mutations [3,4] and creation of organisms with specific genetic backgrounds [5,6,7]. The system requires Cas9 protein and a guide RNA (gRNA) that specifically recognizes the target sequence in a genomic DNA [1]. The Cas9 and gRNA complex causes a site-specific DNA double-strand break (DSB) on the target sequence. DNA DSBs are repaired by two major pathways: non-homologous end-joining (NHEJ) and homology-directed repair (HDR) [8,9]. In the NHEJ pathway, gene knockout due to random insertion–deletion (indel) mutations is expected, and the sequence after indel mutations is unpredictable. In the HDR pathway, DNA DSBs are precisely repaired using an exogenous donor DNA template. By supplying a single-stranded oligodeoxynucleotide (ssODN) template along with Cas9 and gRNA into cells, editing via accurate HDR can be achieved [10,11]. Although genome editing via HDR is desired in most applications where accurate editing is required, its use is limited because of its low efficiency [12,13].

A versatile and effective strategy to increase HDR efficiency with minimal manipulation of the cell is to localize a high concentration of donor DNA at the DSB site. This is because the presence of donor DNA in the immediate vicinity of the DSB site is critical for repairing DNA via HDR, rather than other repair pathways [14]. Therefore, simultaneous delivery of donor ssODNs to the target site has been attempted by combining ssODNs with Cas9 or gRNA [14,15,16,17,18,19,20]. The integration of donor ssODNs into CRISPR components is also effective in improving the efficiency of intracellular introduction. This is because it eliminates the need to introduce individual ssODNs into the cell and reduces the difficulty of the simultaneous introduction of multiple types of molecules into the same single cell. In previous reports, Cas9 was bound to ssODNs via fused domains [15,16] or substrates [14,17,18,19,20], achieving increased HDR efficiency without altering the endogenous cellular repair process. However, fusion of additional domains to Cas9 may affect its expression and stability. Moreover, complex engineering of Cas9 proteins and chemical modification of ssODNs are cumbersome and costly, leading to barriers to their widespread use. In addition, because of the difficulty of direct protein introduction into cells through cell walls [21,22], in vitro conjugation of ssODNs and Cas9 proteins before intracellular introduction is not suitable for organisms such as plants and fungi. 

In this study, we created a donor DNA/gRNA hybrid duplex (DGybrid) that functions as both a gRNA and donor DNA in a single molecule. This novel DGybrid is composed of a sequence-extended gRNA and unmodified ssODNs, which are combined with complementary bases without chemical modifications. Genome editing using DGybrid successfully improved the editing efficiency via HDR by 1.8-fold (from 35% to 62%) in *Saccharomyces cerevisiae* without constitutive expression of the gRNA. This novel strategy would be the most economical, simple, and universal tool for efficient HDR-mediated editing of genomic DNA.

## 2. Materials and Methods

### 2.1. Strains and Media

*Escherichia coli* strain DH5α (F*^−^*, Φ80d*lacZ*ΔM15, Δ(*lacZYA*-*argF*)U169, *deoR*, *recA1*, *endA1*, *hsdR17* (r*_k_*^−^, m*_k_*^+^), *phoA*, *supE44*, λ^−^, *thi-1*, *gyrA96*, *relA1*) (Toyobo, Osaka, Japan) was used as the host for recombinant DNA manipulation. Transformed DH5α cells were grown in LB + A medium (1% (*w*/*v*) tryptone, 0.5% (*w*/*v*) yeast extract, and 1% (*w*/*v*) sodium chloride) containing 100 μg/mL ampicillin. *S. cerevisiae* BY4741 (*MAT* **a**, *his3Δ1*, *leu2Δ0*, *met15Δ0*, *ura3Δ0*) was used as the host strain for genome editing. Yeast cells were cultured in the following media: YPD medium (1% (*w*/*v*) yeast extract, 2% (*w*/*v*) peptone, and 2% (*w*/*v*) glucose); SDC medium (0.67% (*w*/*v*) yeast nitrogen base without amino acids, 2% (*w*/*v*) glucose, and 0.5% (*w*/*v*) casamino acids); SD + HLM medium (0.67% (*w*/*v*) yeast nitrogen base without amino acids, 2% (*w*/*v*) glucose, 0.002% (*w*/*v*) histidine, 0.003% (*w*/*v*) leucine, and 0.003% (*w*/*v*) methionine); SD + HLM with canavanine medium (0.67% (*w*/*v*) yeast nitrogen base without amino acids, 2% (*w*/*v*) glucose, 0.002% (*w*/*v*) histidine, 0.003% (*w*/*v*) leucine, 0.003% (*w*/*v*) methionine, and 0.001% (*w*/*v*) l-canavanine sulfate (Nacalai Tesque, Kyoto, Japan)).

### 2.2. Construction of Plasmids

All primers used for plasmid construction are listed in Table S1. All DNA fragments were amplified by polymerase chain reaction (PCR) using KOD-One DNA polymerase (Toyobo). Fusion of the Cas9 gene from *Streptococcus pyogenes* and the nuclear localization signal of the SV40 large T antigen was amplified from p414-TEF1p-Cas9-CYC1t [10] using primers (Cas9-F and Cas9-R). Amplified Cas9_NLS was inserted into p416 GPD [23], which was linearized by amplification using primers (p416GPD-F and p416GPD-R). Insertion was performed using an In-Fusion HD Cloning kit (Takara Bio, Otsu, Japan). The resulting plasmid was named p416GPD_Cas9_NLS. The plasmid sequences were confirmed using Sanger sequencing.

### 2.3. Yeast Transformation

The plasmid p416GPD_Cas9_NLS was introduced into yeast using the Frozen-EZ Transformation Kit II (Zymo Research, CA, USA). The transformants were selected on SDC solid medium.

### 2.4. In Vitro Synthesis of gRNA

gRNA and other extended gRNAs, which are necessary for DGybrid preparation, were synthesized using the CUGA^®^7 gRNA Synthesis Kit (NIPPON GENE, Tokyo, Japan). DNA templates with T7 promoter and gRNA sequences were prepared from three oligonucleotide primers by overlap extension PCR using KOD-One DNA polymerase and then purified using the QIAEX Gel Extraction Kit (Qiagen, Hilden, Germany). All primers used for preparing gRNA template are listed in Table S1. Furthermore, gRNA synthesis was performed according to the manufacturer’s protocol. The synthesized gRNAs were purified using a spin column provided with the CUGA^®^7 gRNA Synthesis Kit.

### 2.5. Preparation of DGybrid

Extended gRNA was synthesized as described previously. The gRNA and ssODN sequences are listed in Table S2. Equimolar amounts of each complementary extended gRNA and ssODNs were mixed in TE buffer with salt (10 mM Tris-HCl (pH 8.0), 1 mM EDTA, and 10 mM NaCl). DGybrid was formed by incubating the mixture at 95 °C for 5 min and then cooling slowly to 25 °C. 

### 2.6. Native-PAGE Analysis of DGybrid

DGybrid formation was confirmed by size fractionation using 10% native-PAGE. For evaluation of formation, DGybrid was formed from 150 pmol each of extended gRNA and ssODNs by the method described above. The samples were mixed with equal volumes of native loading buffer (30% (*v*/*v*) glycerol, 80 mM HEPES-KOH (pH 7.9), 100 mM KCl, and 2 mM magnesium acetate) and electrophoresed in 1× TBE buffer at 100 V and 25 °C for 80 min in a vertical gel tank. Dilutions of each gRNA and ssODN in RNase-free water were also performed to calculate the efficiency of hybrid formation. Moreover, 1 kb Plus DNA Ladder (New England BioLabs, Ipswich, MA, USA) was loaded as a reference for nucleic acid size. The DGybrid and its counterparts were stained with ethidium bromide and visualized using a UV transilluminator. Band intensities were quantified with ImageJ software (ver.1.53e; National Institute of Health, MS, USA).

### 2.7. Introduction of DGybrid into Cells by Electroporation

For genome editing, DGybrid was transformed into BY4741 cells harboring the p416GPD_Cas9_NLS plasmid by electroporation. The DGybrid used for genome editing was formed from 200 pmol of each of the extended gRNA and ssODNs by the method described above. First, BY4741 cells harboring the p416GPD_Cas9_NLS plasmid were grown overnight in 10 mL SDC liquid medium. Cells that reached the stationary phase were inoculated into 100 mL of YPD liquid medium to obtain a starting OD_600_ of 0.2 and grown to an OD_600_ of 1.4~1.7. The cells were collected by centrifugation at 900× *g* for 5 min. The collected cells were washed twice with sterilized water and once with electroporation buffer (1 M sorbitol and 1 mM CaCl_2_). The cells were then resuspended in 100 mM LiAc and 10 mM DTT solution and incubated at 30 °C for 30 min with shaking at 230 rpm. After washing again with electroporation buffer, the cells were resuspended in electroporation buffer to a final volume of 1.0 mL. Cell suspensions (300 µL) were mixed with 200 pmol/30 µL of DGybrid or other nucleic acid solutions and electroporated once at 2500 V in a 0.2 cm cuvette. The electroporated cells were quickly transferred to 4 mL of a 1:1 mixture of 1 M sorbitol and YPD medium. The cells were incubated at 30 °C for 2 h with shaking at 230 rpm.

### 2.8. Evaluation of Genome-Editing Efficiency

After incubation of the electroporated cells for 2 h, the cells were inoculated into 10 mL of SDC liquid medium to obtain an OD_600_ of 0.1 and grown for 24 h. Then, the cells were collected and diluted to an OD_600_ of 0.001. Diluted cultures (100 µL) were spread onto SD + HLM solid medium or SD + HLM with canavanine solid medium. After incubation at 30 °C for 72 h, images of each solid medium were captured, and the number of colonies was counted using ImageJ software (ver.1.53e). Genome-editing efficiency was evaluated by the rate of appearance of canavanine-resistant colonies relative to the total number of cells identified in the canavanine-free medium.

### 2.9. Evaluation of Introduction Efficiency of Nucleic Acids into Cells Using Flow Cytometry

To evaluate the efficiency of electroporation-based introduction of nucleic acids into yeast cells, fluorescently labeled nucleic acids (Table S2) were used. Fluorescently labeled DNA/RNA hybrids were formed from 60 pmol each of RNA_60 and ssODN_60 using the method described above, whose 3′ end was labeled with Alexa Fluor 647 (AF647) and fluorescein isothiocyanate (FITC), respectively. ssODN_20, which was also labeled with FITC at the 3′ end, lacks the sequence for annealing to RNA_60 and was used as a control. AF647-labeled RNA was purchased from Ajinomoto Bio-Pharma Services (Tokyo, Japan). FITC-labeled DNA was purchased from Eurofins Genomics (Tokyo, Japan).

A fluorescently labeled DNA/RNA hybrid was introduced into BY4741 cells harboring the p416GPD_Cas9_NLS plasmid by electroporation. For electroporation, nucleic acids (20 pmol/3 µL) were introduced into 30 µL of the cell suspension. Electroporated cells were incubated in a 1:1 mixture of 1 M sorbitol and YPD medium at 30 °C for 10 min with shaking at 230 rpm and then collected at 3500× *g* for 2 min. The cells were washed twice with PBS (pH 7.4) and resuspended in PBS. The fluorescence intensity of the cells was measured using flow cytometry (JSAN; Bay Bioscience, Kobe, Japan), and the introduction efficiency was evaluated based on the percentage of fluorescent cells. The fluorescence of AF647 was detected with excitation at 640 nm and emission at 661 ± 10 nm (FL5), and FITC was detected with excitation at 488 nm and emission at 535  ±  23 nm (FL1). The fluorescence intensities of 20,000 yeast cells were plotted as a density plot. Data were analyzed using Kaluza software (ver. 2.1; Beckman Coulter, Brea, CA, USA). In the density plot, the ratios of yeast cells that richly took up the nucleic acids were quantified.

## 3. Results

### 3.1. Preparation of DGybrid

**Figure 1:**
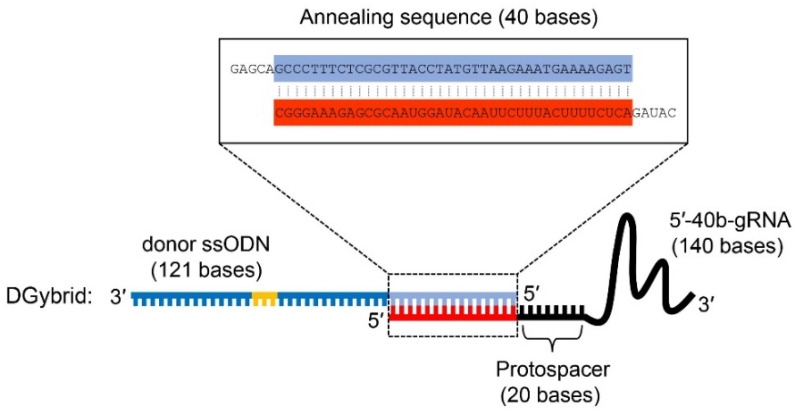
Design of donor DNA/guide RNA (gRNA) hybrid duplex (DGybrid). DGybrid was formed by the hybridization of donor single-stranded oligodeoxynucleotide (ssODN) and 5′-40b-gRNA via annealing sequence (40 bases). For ssODN, the blue- and light-blue-colored sequences show homologous sequences to the target gene. The light-blue-colored sequence was also used for annealing with 5′-40b-gRNA. The yellow-colored bases show an introduced mutation. For 5′-40b-gRNA, the black- and red-colored sequences show the conventional gRNA sequence and extended sequence for annealing, respectively.

To achieve higher HDR efficiency in CRISPR-based genome editing, we attempted to develop a novel tool that combines donor ssODNs and gRNA through base pairing between complementary DNA and RNA sequences. For this purpose, we designed a unique gRNA (5′-40b-gRNA), in which the 5′ end of a conventional gRNA is extended by 40 bases complementary to the 5′ end of the donor ssODN (Figure 1). Compared to the 3′ end, the 5′ end of gRNA is known to be more tolerant to modification [20,24]. Furthermore, ssODNs (121 bases) were used as donor DNA. When 5′-40b-gRNA and ssODNs are mixed in equal molar amounts, their complementary 40 nucleotide sequences form base pairs, producing a donor DNA/gRNA hybrid duplex (DGybrid) that has both gRNA and donor DNA functions.

**Figure 2:**
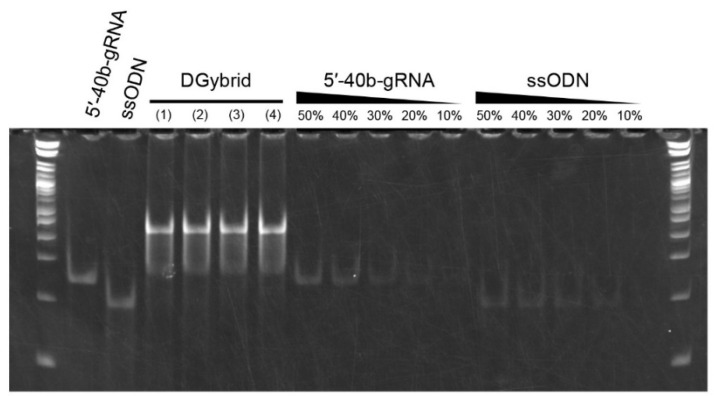
Native-PAGE analysis to confirm DGybrid formation. Values above the lanes of 5′-40b-gRNA and ssODN indicate the relative molar amounts of the applied sample. In the two lanes at both ends, 1 kb Plus DNA Ladder was applied. The DGybrid samples were prepared in four different conditions: (1) TE buffer, (2) TE buffer + 10 mM NaCl, (3) TE buffer + 100 mM NaCl, and (4) ethanol precipitation of the sample prepared in condition (3).

We performed native-PAGE analysis to examine DGybrid formation. The DGybrid samples were prepared in four different conditions: (1) TE buffer, (2) TE buffer + 10 mM NaCl, (3) TE buffer + 100 mM NaCl, and (4) ethanol precipitation of the sample prepared in condition (3). In the four lanes to which the formed DGybrids were applied, the bands corresponding to 5′-40b-gRNA and ssODNs almost completely disappeared. Instead, a new intense band was observed on the high-molecular-weight side at the top of the gel (Figure 2), indicating the formation of DGybrid. In addition, based on the band intensities of the diluted solutions of 5′-40b-gRNA and ssODNs, the amount of both counterparts remaining in the DGybrid sample prepared under condition (2) is less than 20%. This indicates that more than 80% of the 5′-40b-gRNA and ssODN contributed to the hybrid formation in the DGybrid sample prepared under condition (2).

### 3.2. Genome Editing via HDR with DGybrid

We evaluated whether DGybrid can achieve HDR-mediated editing with higher efficiency than conventional methods. DGybrid was introduced into *S. cerevisiae* constitutively expressing Cas9 from the p416GPD_Cas9_NLS plasmid, and the editing efficiency was evaluated based on the phenotype (canavanine resistance). The DGybrid was designed to target the *CAN1* gene as a function of gRNA and to cause nonsense mutations in the PAM sequence as a function of donor DNA. When a stop codon is placed in the *CAN1* gene by HDR-mediated editing, the function of Can1 (arginine transporter) is abolished, and yeast cells acquire improved canavanine resistance [25,26].

**Figure 3:**
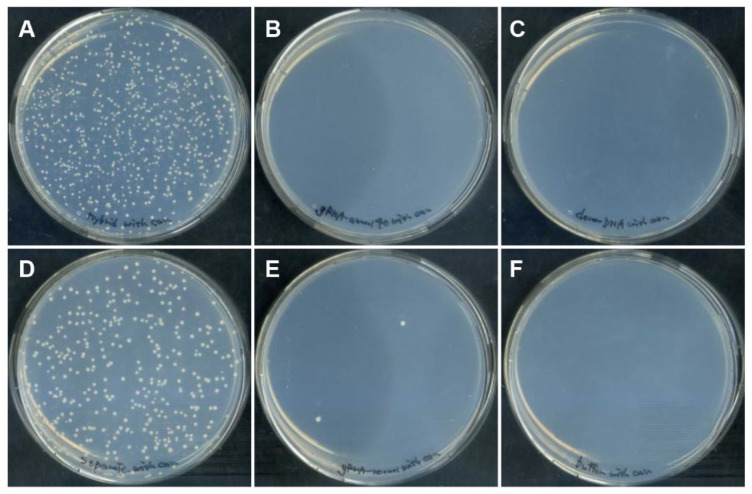
Colony formation on the canavanine-containing medium after DGybrid introduction. Yeast cells were subject to electroporation of the nucleic acids; (**A**) DGybrid, (**B**) 5′-40b-gRNA only, (**C**) ssODN only, (**D**) gRNA and ssODN, (**E**) gRNA only, and (**F**) neither gRNA nor ssODN.

**Figure 4:**
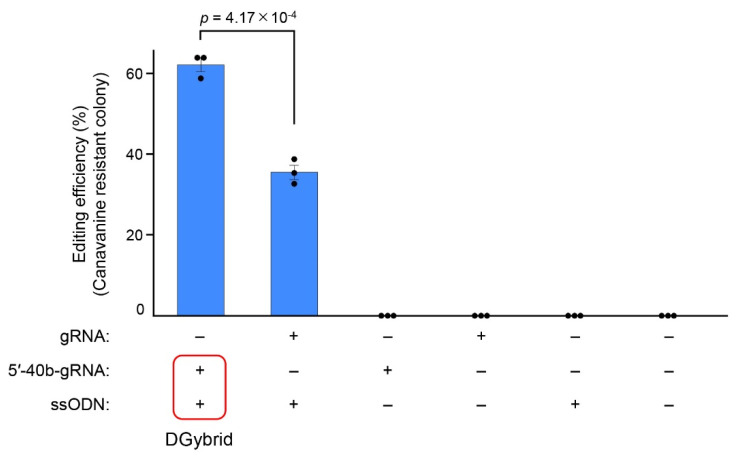
Evaluation of the DGybrid-based genome-editing efficiency. The genome-editing efficiency was evaluated by counting the number of canavanine-resistant colonies. Error bars represent the SEM of three biological replicates starting from independent electroporation of nucleic acid solutions. Points represent each experimental data set. A two-tailed Student’s *t*-test was used to assess the statistical significance.

Cells introduced with DGybrid were inoculated on an agar medium containing canavanine, and resistant colonies were obtained (Figure 3). The results show that the genome-editing efficiency of the sample using DGybrid (62%) was significantly higher than that of the conventional method (35%), in which gRNA and donor ssODNs worked separately (Figure 4). Sanger sequencing of the *CAN1* gene from the obtained canavanine-resistant colonies showed that the stop codon was inserted precisely at the target site (Figure S1). These results indicate that the developed DGybrid can achieve precise HDR-mediated editing more efficiently than conventional methods.

The CRISPR/Cas9 system, which induces DSBs, is potentially cytotoxic. To investigate the effect of DGybrid on cytotoxicity, we monitored the growth of yeast cells introduced with DGybrid by measuring OD_600_. The growth of DGybrid-introduced cells was not significantly different from that of cells in which donor ssODN and gRNA were introduced separately (Figure S2). Therefore, it is suggested that the DGybrid would not affect CRISPR toxicity.

Potentially, the length of the annealing sequence between gRNA and donor ssODNs may affect the stability of the DGybrid and HDR-mediated editing efficiency in yeast cells. Thus, the effect of DGybrid design on HDR-mediated editing efficiency was examined by extending the length of the annealing sequence to 70 bases (Figure S3A). In the first case (DGybrid 70-A), the annealing sequence was extended to 70 bases, but the homologous sequence was correspondingly reduced to 51 bases. In the second case (DGybrid 70-B), the annealing sequence was similarly extended to 70 bases, and 81 bases of the homologous sequence were used, as in the original design. These modified DGybrid showed editing efficiencies comparable to those of the initial DGybrid (Figure S3B). These results suggest that, regardless of the length of the annealing sequence, the combination of donor ssODNs and gRNA into a single unit is critical for improving editing efficiency.

### 3.3. Properties of DNA/RNA Hybrid Contributing to Improved Editing Efficiency

Our DGybrid successfully improved the HDR-mediated editing efficiency in yeast cells. We assumed that the improved HDR-mediated editing efficiency could be due to the following two advantageous properties of the DGybrid: First, the donor DNA can be localized near the cleavage site using DGybrid. The donor ssODN can be recruited to the cleavage site by combining it with the gRNA that recognizes the target sequence as reported in some previous studies [14,15,16,17,18,19,20]. Thus, ssODNs are quickly available before the cleavage site is subjected to repair mechanisms such as NHEJ or degradation other than HDR, leading to a positive effect on editing efficiency. Second, DGybrid has the advantage of introduction when incorporating gRNA and donor DNA into a cell. When two oligonucleotides are introduced into a cell separately using the conventional method, the efficiency of the cells in taking up both oligonucleotides is low. Using DGybrid, both gRNA and donor ssODNs can be delivered together into the cell. Thus, the cell obtains both donor ssODNs and gRNA with only one molecule uptake and more HDR-mediated editing opportunities. This can also lead to improvements in editing efficiency. 

**Figure 5:**
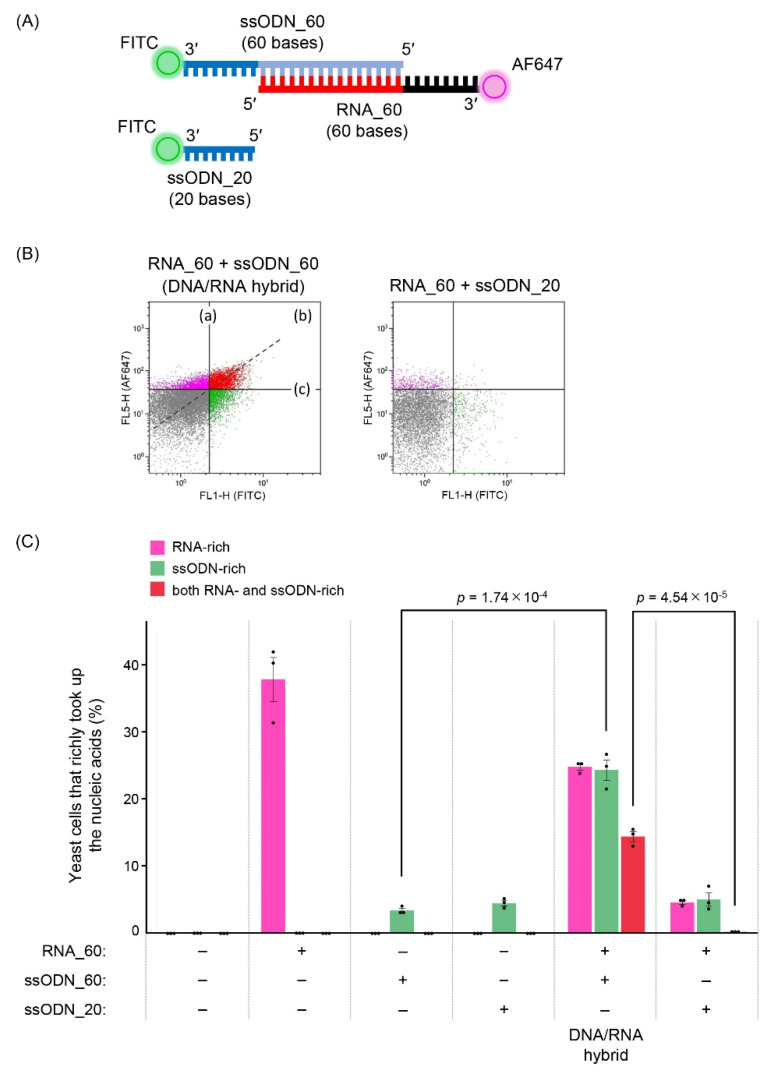
Evaluation of introduction efficiency of DNA/RNA hybrid into yeast cells. (**A**) Schematic of nucleic acids used to evaluate the introduction efficiency. (**B**) Density plots obtained from flow cytometry analysis. (**a**) The ratio of yeast cells that richly took up RNA (RNA-rich yeast cells), (**b**) The ratio of yeast cells that richly took up both RNA and ssODN (both RNA- and ssODN-rich yeast cells), and (**c**) The ratio of yeast cells that richly took up ssODN (ssODN-rich yeast cells). The data shown are representative of three independent experiments. (**C**) The introduction efficiency was evaluated using flow cytometry. Values indicate the ratio of RNA-, ssODN- or both RNA- and ssODN-rich yeast cells. Error bars represent the SEM of three biological replicates starting from independent electroporation of nucleic acid solutions. Points represent each experimental data set. A two-tailed Student’s *t*-test was used to assess the statistical significance.

To assess the contribution of the second advantage, we analyzed the introduction efficiency of the DNA/RNA hybrid into yeast cells. To detect nucleic acids introduced into cells, the 3′ ends of RNA_60 (60 bases) and ssODN_60 (60 bases) were labeled with Alexa Fluor 647 and FITC, respectively (Figure 5A). They were annealed via the same 40-base complementary sequence as DGybrid described in Figure 1, and their successful formation was confirmed by native-PAGE analysis (Figure S4). Flow cytometry analysis of the cells introduced with nucleic acids showed that cells to which the formed DNA/RNA hybrids were introduced were distributed in an approximate linear pattern on the density plot, indicating that the cells took up RNA and ssODN together (Figure 5B). The cell population localized in the upper right of the plot, which took up very large amounts of both RNA and ssODN, was assumed to have more HDR-mediated editing opportunities, and the percentages were compared. In samples to which the DNA/RNA hybrid formed from RNA_60 and ssODN_60 were introduced, 14.4% of the cell populations were rich in both RNA and ssODN. Such a cell population was virtually absent (0.2%) when RNA_60 and ssODN_20 (20 bases), which do not form DNA/RNA hybrids, were introduced together. These results suggest that use of DGybrid also increases the number of cells rich in both gRNA and donor ssODNs, which would contribute to the increased editing efficiency via HDR.

## 4. Discussion

In this study, to increase the recruitment of donor DNA near the cleavage site in the CRISPR/Cas9 system, we created a DGybrid in which gRNA and donor ssODNs were combined by complementary bases. A genome-editing assay in *S. cerevisiae* showed that the *CAN1* gene was precisely edited via HDR with 62% efficiency using a DGybrid, compared to 35% when donor ssODNs and gRNA were used separately (Figure 4). Therefore, DGybrid is a promising tool that improves editing efficiency via HDR. Flow cytometry analysis of the cells introduced with nucleic acids showed that the use of the DNA/RNA hybrid resulted in the efficient uptake of both RNA and ssODN together by the cells (Figure 5B). This advantage resolves the difficulty of introducing multiple kinds of nucleic acids into cells. In conclusion, although there may be a positive effect of the high localization of donor ssODN at the cleavage as claimed in previous studies, the experiments in this paper suggest that the improvement in the efficiency of donor ssODN introduction into cells would be a major contribution.

In the genome-editing assay, the greater number of canavanine-resistant colonies acquired using DGybrid was most likely due to the improved introduction efficiency and the recruitment of donor DNA to cleavage sites. This is suggested by the fact that exogenous donor ssODNs were preferentially used over the abundant endogenous template. Since *S. cerevisiae* has relatively high HDR activity, it seems that DSBs were repaired to an intact state via HDR by utilizing the abundant sister chromatids or homologous chromosomes that were present during the 24 h repeated cell division [27]. Indeed, most yeast cells did not acquire improved canavanine resistance when donor ssODNs were not supplied and only gRNA or 5′-40b-gRNA was supplied (Figure 3 and Figure 4). This finding suggests that the percentage of gene knockout caused by NHEJ was low and that the DSBs were repaired to their original intact state. In cells introduced with DGybrid, HDR with an exogenous donor ssODN as a template was highly efficient, suggesting that the concentration of donor DNA near the cleavage site could be increased enough to overcome the competition with HDR using the endogenous template.

Furthermore, hybridization of DNA and RNA resulted in the efficient uptake of RNA and ssODN together, suggesting that DGybrid could have an advantage in terms of nucleic acid introduction efficiency (Figure 5B). In addition, the percentage of cells that retain abundant ssODNs increased. In samples to which only ssODNs were introduced, the percentages of cells defined as ssODN-rich were 3.4% and 4.5% but increased to 24.3% due to hybrid formation with RNA_60 (Figure 5C and Figure S5). This interesting advantage of DNA/RNA hybrids may be due to the unique physical properties of hybrids, which differ from double-stranded RNA and even double-stranded DNA [28,29] and may have exhibited the advantage in nucleic acid introduction, leading to improved editing efficiency. Further micro-dynamic analysis is required to understand the advantage of DGybrid in intracellular introduction. 

Several approaches for covalently conjugating donor ssODNs to Cas9 or gRNA have been reported [17,18,19,20]. These approaches achieved strong conjugation but required at least chemical modifications to the oligonucleotide or protein, leading to higher costs. In contrast, our DGybrid was formed by complementary bases, and the extended gRNA was synthesized by in vitro transcription at the same cost as regular gRNA. In addition, hybridization was briefly performed by changing the temperature. The sufficient stability of the 40-base pairing used in DGybrid is supported by the fact that there was no difference in editing efficiencies in the DGybrid formed by 40- and 70-base pairing (Figure S3). Thus, our novel DGybrid tool would be the first choice for improving HDR-mediated editing efficiency.

In this study, the advantages of DGybrid were demonstrated in *S. cerevisiae*, but we expect that they can be broadly applied to other organisms. In mammalian cells, the high activity of NHEJ competes with HDR and prevents precise editing [8]. Therefore, DGybrid is useful in promoting DSB repair via HDR. Recently, the use of ribonucleoproteins (RNPs) complexed with Cas9 and gRNA has been favored for genome editing in mammalian cells [30,31]. The transfected RNPs instantly edited the target site and were subsequently degraded, resulting in the suppression of off-target editing outside the target site [31,32]. Complexing the Cas9 protein with a DGybrid is expected to create a single all-in-one tool for genome editing via HDR. This tool would efficiently and promptly perform HDR-mediated editing. In addition, we previously developed a CRISPR Nickase system in which the editable target sites were expanded [26,33]. Compared to the conventional CRISPR/Cas9 system, our CRISPR Nickase system provides precise genome editing on expanded target sites without off-target editing, but the absolute efficiency of HDR-mediated genome editing is reduced. This lower efficiency could be improved by applying DGybrid to the CRISPR/Nickase system.

In conclusion, we developed DGybrid that can efficiently recruit donor DNA close to the cleavage site by hybridizing donor ssODNs and gRNA via complementary bases. This tool would be a useful option for improving HDR-mediated editing efficiency and can be used consistently and conveniently across a wide range of organisms.

## Data Availability

The data underlying this study are available in the article and online Supplementary Materials.

## Supplementary Materials

The following are available online at https://www.mdpi.com/article/10.3390/biom12111621/s1: Figure S1: Sequencing analysis of the target sequence of the *CAN1* gene edited by DGybrid-based genome editing, Figure S2: Growth of Cas9-expressing yeast cells electroporated with nucleic acid solutions, Figure S3: Evaluation of the effect of various DGybrid designs on genome-editing efficiency, Figure S4: Native-PAGE analysis to confirm DNA/RNA hybrid formation, Figure S5: Density plots obtained from flow cytometry analysis. Table S1: List of primers used in this study, Table S2: List of RNA and ssODNs used in this study.
