# Supplementary material for: Improving Precise Genome Editing Using Donor DNA/gRNA Hybrid Duplex Generated by Complementary Bases
Source: Biomolecules. 2022 Nov 3;12(11):1621. doi: 10.3390/biom12111621 (PMC9687273; doi:10.3390/biom12111621)
Supplement: Supplementary file 1 [file biomolecules-12-01621-s001.zip › biomolecules-1944754-supplementary/Supplementary_Figure_Aiba_et_al.pdf]

	5′ gRNA-targeting sequence		PAM 3′
Reference	GATACGTTCT	CTATGGAGGA	TGGCATAGGT
DGybrid(7/7)	GATACGTTCT	CTATGGAGGA	TTAAATAGGT
gRNA + ssODN(7/7)	GATACGTTCT	CTATGGAGGA	TTAAATAGGT
gRNA (1/2)	GATACGTTCT	C---------A	TGGCATAGGT
gRNA (1/2)	GATACGTTCT	CTATGGAAGGA	TGGCATAGGT

Figure S1. Sequencing analysis of the target sequence of the *CAN1* gene edited by DGybrid-based genome editing. The red-colored and underlined bases show the introduced stop codons, and the red-colored bases show unintended mutation or deletion. The numbers of observed sequences over the numbers of total sequenced strains (e.g., 4/4) are shown.

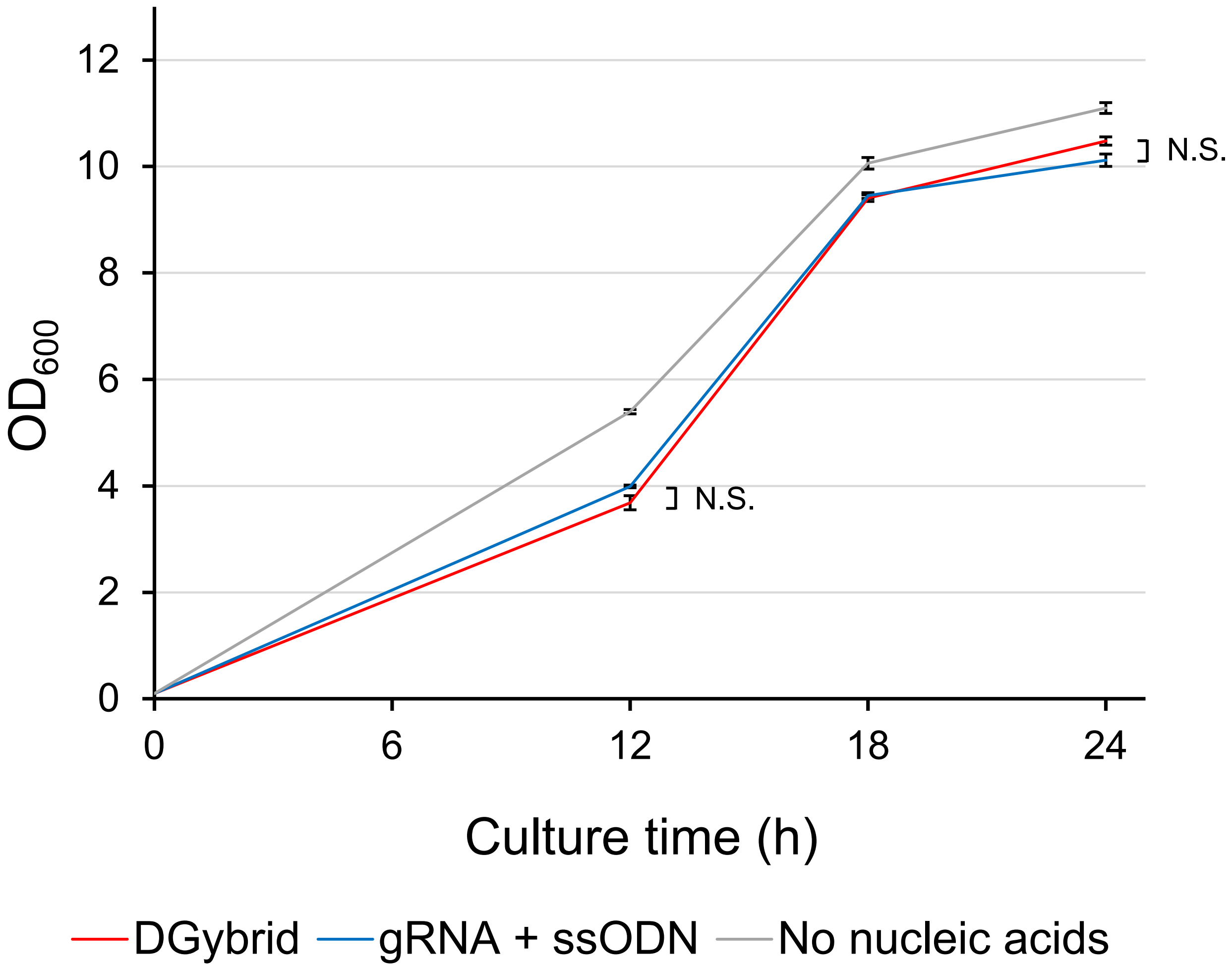

Figure S2. Growth of Cas9-expressing yeast cells electroporated with nucleic acid solutions. After incubation of the electroporated cells for 2 h, the cells were inoculated into 10 mL of SDC liquid medium to obtain an OD_{600} of 0.1. Thereafter, cell growth was monitored by measuring absorbance at 600 nm. Error bars represent the SEM of three biological replicates starting from independent electroporation of nucleic acid solutions. A two-tailed Student's *t*-test was used to assess the statistical significance.

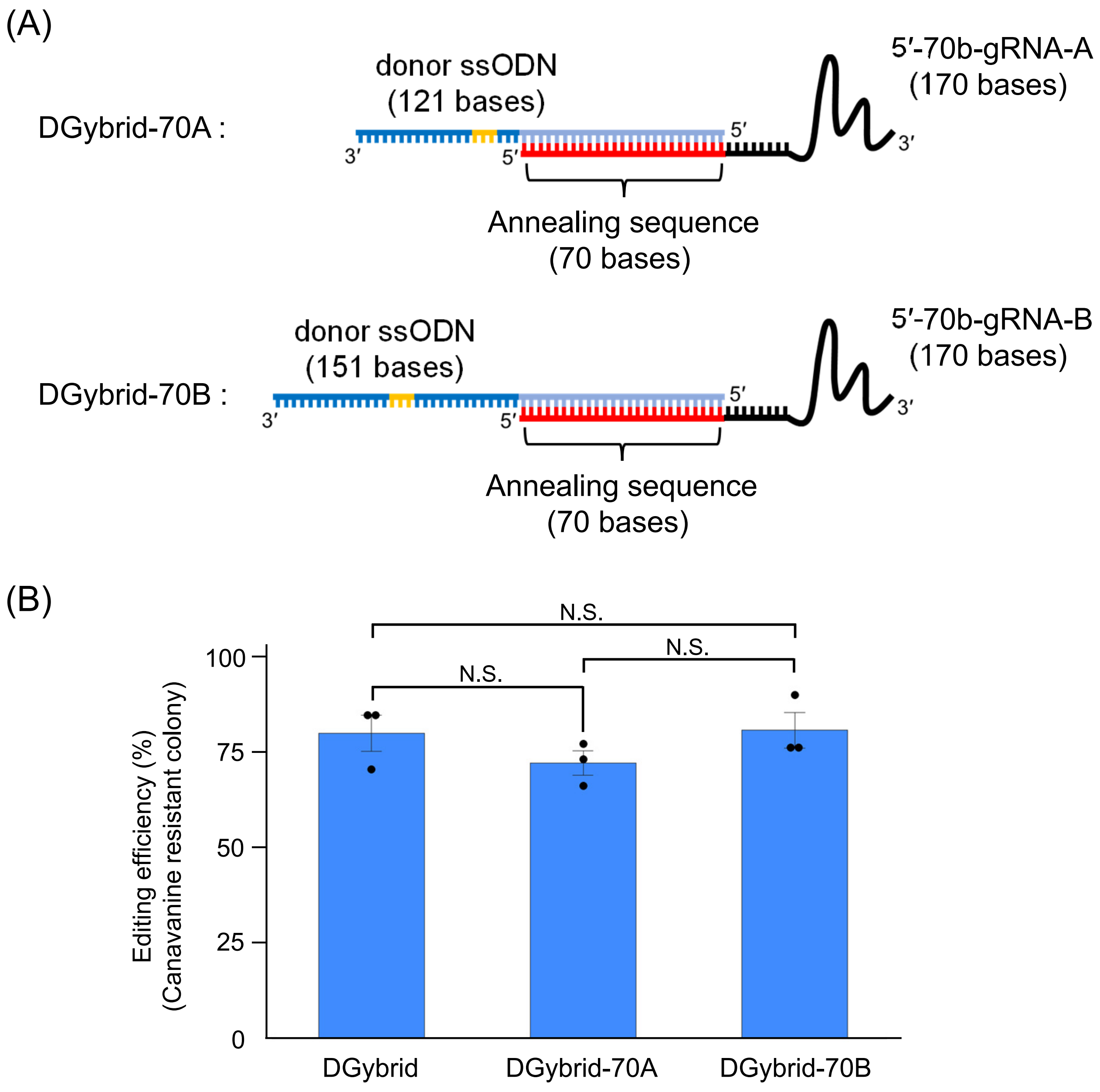

Figure S3. Evaluation of the effect of various DGybrid designs on genome-editing efficiency. (A) Design of DGybrid with different lengths of base pair formation. The ssODN (121 bases) and ssODN (151 bases) (blue) are shown with the mutation position (yellow). For 5'-70b-gRNA-A and 5'-70b-gRNA-B, the black- and red-colored sequences show the conventional gRNA sequence and extended sequence, respectively. Through the complementary sequence (70 bases), ssODN (121 bases) and ssODN (151 base) bind to 5'-70b-gRNA-A and 5'-70b-gRNA-B, respectively. (B) Comparison of genome-editing efficiencies between various designs of DGybrid. The genome-editing efficiency was evaluated by counting the number of canavanine-resistant colonies. Error bars represent the SEM of three biological replicates starting from independent electroporation of nucleic acid solutions. Points represent each experimental data. A two-tailed Student's *t*-test was used to assess the statistical significance.

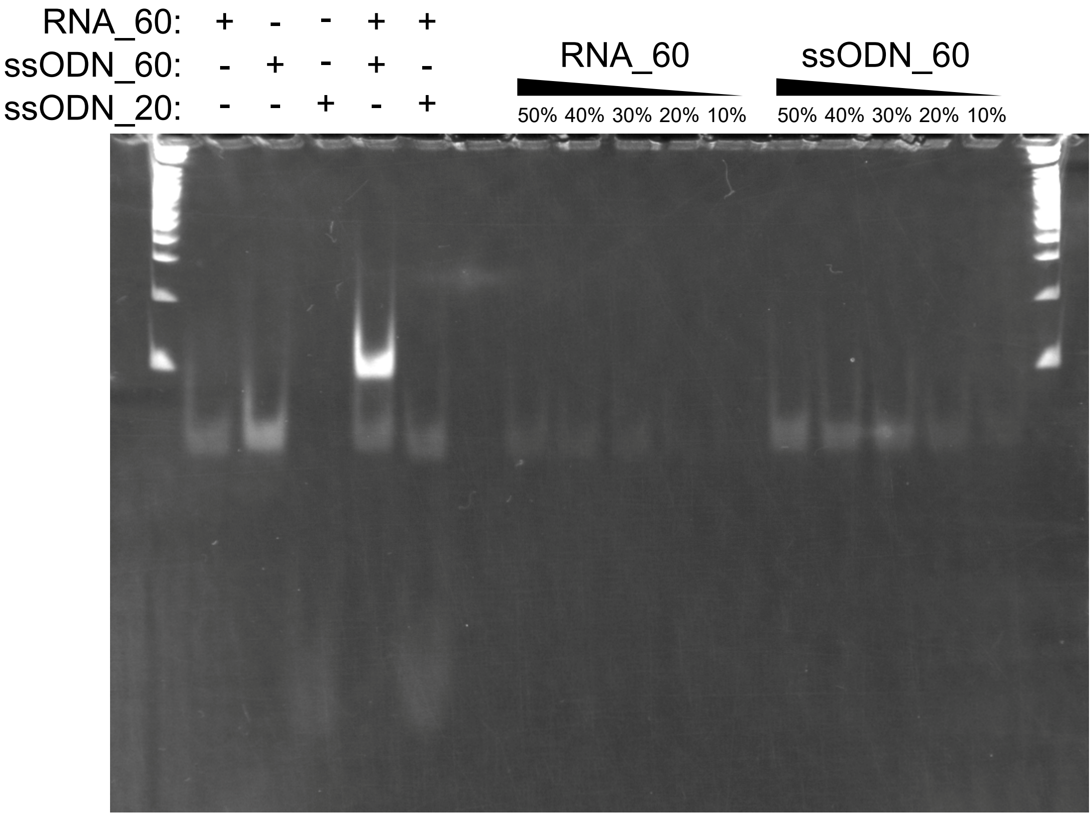

Figure S4. Native PAGE analysis to confirm DNA/RNA hybrid formation. Values above the image indicate the relative molar amounts of the applied samples. In the two lanes at both ends, 1 kb Plus DNA ladder was applied.

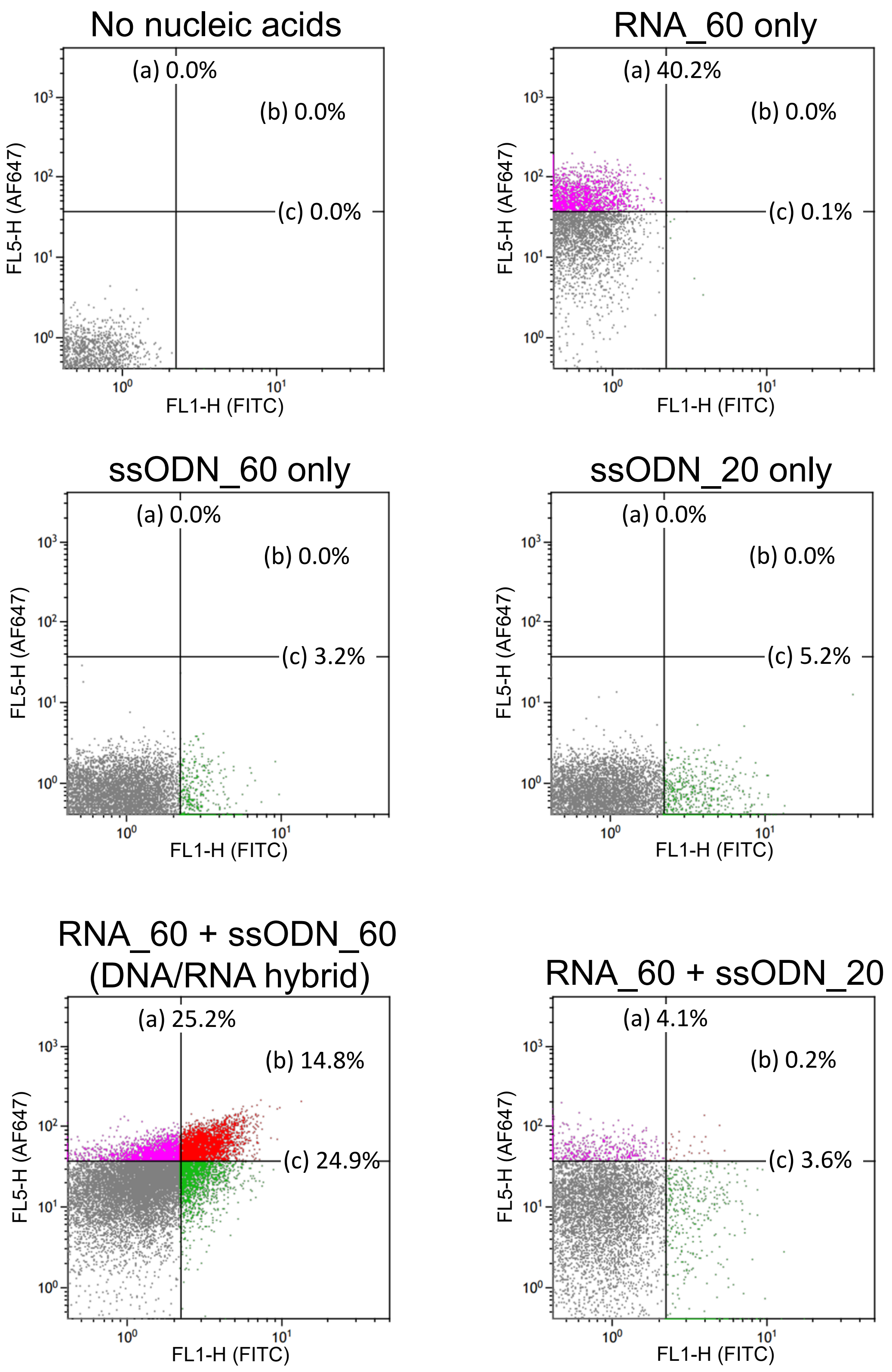

Figure S5. Density plots obtained from flow cytometry analysis. (a) The ratio of RNA-rich yeast cells, (b) ratio of yeast cells both RNA- and ssODN-rich, and (c) ratio of ssODN-rich yeast cells. The data shown are representative of three independent experiments.
